# Stimulation of TRPA1 attenuates ischemia-induced cardiomyocyte cell death through an eNOS-mediated mechanism

**DOI:** 10.1080/19336950.2019.1623591

**Published:** 2019-06-04

**Authors:** Spencer R. Andrei, Monica Ghosh, Pritam Sinharoy, Derek S. Damron

**Affiliations:** a Department of Medicine, Vanderbilt University Medical Center, Nashville, TN, USA; b Department of Biomedical Sciences, Kent State University, Kent, OH, USA; c Department of Biopharmaceutical Development, Medimmune LLC, Gaithersburg, MD, USA

**Keywords:** TRPA1, nitric oxide, cardiomyocytes

## Abstract

The functional expression of transient receptor potential cation channel of the ankyrin-1 subtype (TRPA1) has recently been identified in adult mouse cardiac tissue where stimulation of this ion channel leads to increases in adult mouse ventricular cardiomyocyte (CM) contractile function via a Ca^2+^-Calmodulin-dependent kinase (CaMKII) pathway. However, the extent to which TRPA1 induces nitric oxide (NO) production in CMs, and whether this signaling cascade mediates physiological or pathophysiological events in cardiac tissue remains elusive. Freshly isolated CMs from wild-type (WT) or TRPA1 knockout (TRPA1^-/-^) mouse hearts were treated with AITC (100 µM) and prepared for immunoblot, NO detection or ischemia protocols. Our findings demonstrate that TRPA1 stimulation with AITC results in phosphorylation of protein kinase B (Akt) and endothelial NOS (eNOS) concomitantly with NO production in a concentration- and time-dependent manner. Additionally, we found that TRPA1 induced increases in CM [Ca^2+^]_i_ and contractility occur independently of Akt and eNOS activation mechanisms. Further analysis revealed that the presence and activation of TRPA1 promotes CM survival and viability following ischemic insult via a mechanism partially dependent upon eNOS. Therefore, activation of the TRPA1/Akt/eNOS pathway attenuates ischemia-induced CM cell death.

## Introduction

The transient receptor potential channels of the ankyrin-1 subtype (TRPA1) are non-selective cation channels that are extensively expressed in sensory neurons [1–3] where they characteristically exhibit high permeability to calcium [4]. Recently, TRPA1 has emerged as a target of interest in the regulation of cardiovascular physiology and pathophysiology [5–7]. Our lab previously identified that TRPA1 is expressed in murine cardiac muscle throughout the endocardium, myocardium and epicardium and colocalizes with TRPV1 at the costameres, z-discs and intercalated discs in the cardiomyocytes (CM) [8]. The activation of TRPA1, as well as TRPV1, results in a transient, dose-dependent increase in intracellular free Ca^2+^ concentration ([Ca^2+^]_i_) [8]. Moreover, our lab has recently demonstrated that TRPA1 stimulation of freshly isolated CMs enhances CM contractile function via a CaMKII-dependent pathway leading to increases in [Ca^2+^]_i_ with a marked enhancement in the timing parameters of the Ca^2+^ transient and the CM contractile response [9]. Additionally, recent studies demonstrate that stimulation of TRPA1 limits injury following cardiac ischemia [10]. Although a few downstream signaling mediators of TRPA1 in cardiac cells have been identified, the precise cellular signal transduction pathways and molecular mechanism(s) in which the ion channel is involved in modulating physiological or pathophysiological events in the heart remains to be fully elucidated.

Nitric oxide (NO) is expressed ubiquitously in almost all major tissue types in the body where it mediates a myriad of cellular physiological and pathophysiological events [11,12]. NO is primarily synthesized by the three different nitric oxide synthase (NOS) isoforms: inducible (iNOS), neuronal (nNOS) and endothelial NOS (eNOS). The role of eNOS in systemic vascular endothelial cells has been well established; however, it is also rapidly becoming an attractive target of interest in the regulation of cardiac physiology [12,13]. eNOS has been implicated in serving a variety of roles within cardiac tissue including mediating the cardiac inotropic response to sustained stretch [14] and myocardial protection following ischemic insult [15,16]. Furthermore, post-translational modification of serine 1177 in eNOS (eNOSpS^1177^) has been implicated as an indicator of enzyme activation [17] that can be modulated by Akt/PKB [18,19] signaling pathways which mediate the enzymes activity and intracellular localization of eNOS [20]. Akt activation mechanisms are of significant interest in cardiac tissue as well where stimulation of Akt (indicated by phosphorylation at serine 473; AktpS^473^) has been implicated in protection following myocardial ischemia [21], mediating the apoptotic response [22], as well as modulating several other physiological events in myocardial tissue [23,24]. The polymodal activation mechanisms and intricate regulatory functions of Akt, eNOS and NO suggest a notable complexity of the cellular signal transduction pathways in which they are involved. Indeed, furthering our understanding of the processes by which Akt and eNOS are activated and NO production is induced in cardiac tissue is of great translational significance.

Based on the previous investigations implicating TRPA1 downstream signaling mediators involving CaMKII in CM contractile function, we hypothesized that the TRPA1 agonist, allyl isothiocyanate (AITC), enhances CM contractile function through an Akt/eNOS-dependent mechanism. We found that TRPA1 stimulation induces phosphorylation of Akt at serine 473 and eNOS at serine 1177 in freshly isolated CMs concomitantly with NO production. However, we demonstrate that, although Akt and eNOS do not play a role in the TRPA1-induced increases in CM [Ca^2+^]_i_ and contractile function, eNOS appears to mediate the TRPA1-elicited attenuation of ischemia-induced cardiac cell death.

## Results

### TRPA1 stimulation with AITC elicits Akt and eNOS phosphorylation concomitantly with NO production in a time-dependent manner in CMs

**Figure 1.**
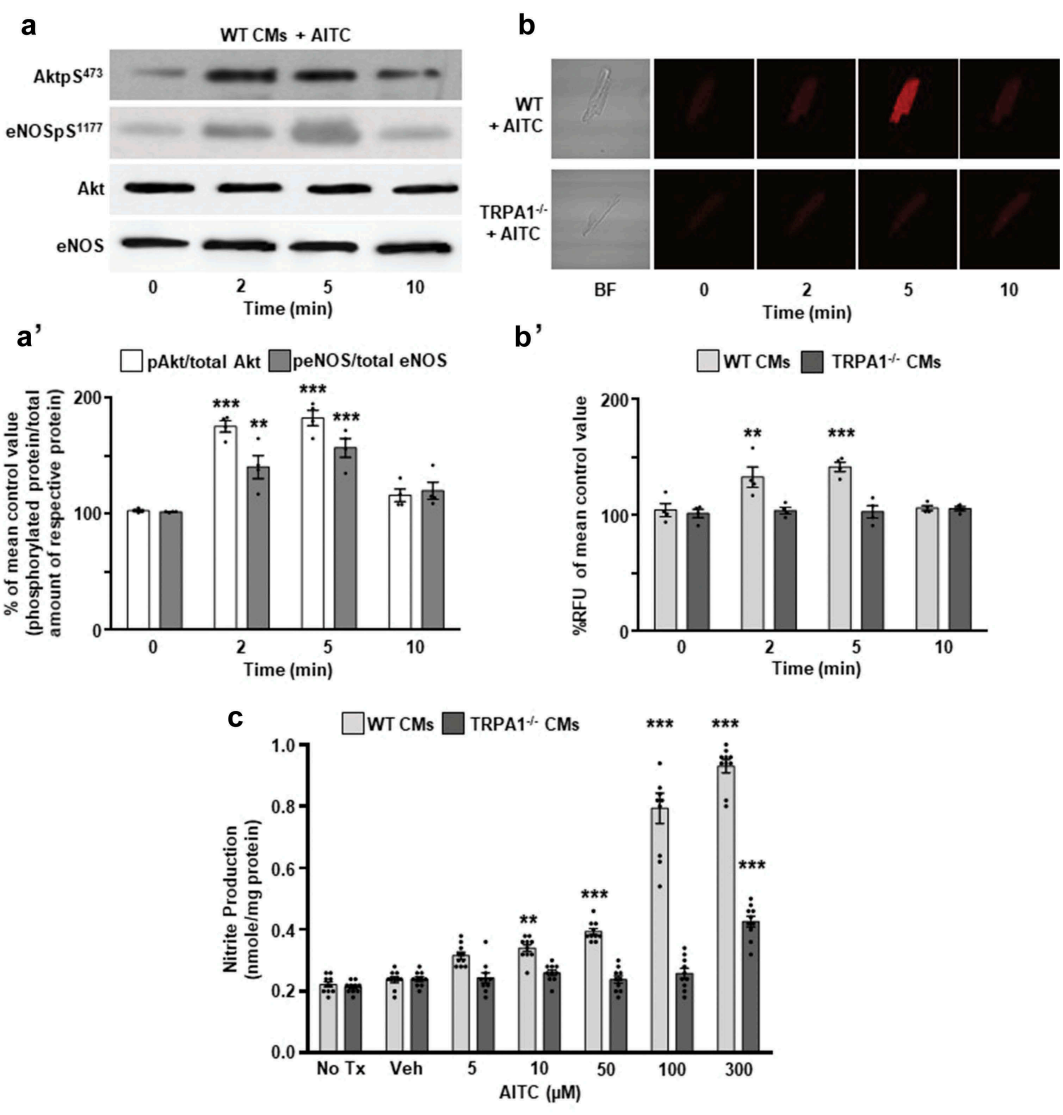
TRPA1 stimulation elicits Akt and eNOS phosphorylation concomitantly with nitric oxide (NO) production in CMs. (a) Representative immunoblots and (b) fluorescent images depicting the time-dependent effects of AITC (100 µM) on Akt serine 473 and eNOS serine 1177 phosphorylation (AktpS^473^ and eNOSpS^1177^, respectively) and NO production in wild-type (WT) and TRPA1 knockout (TRPA1^-/-^) cardiomyocytes (CMs) measured at 0, 2, 5, 10 min. Total Akt and eNOS were probed as loading controls for immunoblots. (A’) Summarized data for Figure 1(a) and (B’) Figure 1(b). Immunoblot data are expressed as a percent of the untreated mean control values (phosphorylated protein/total amount of respective protein) ± SEM, whereas NO data are expressed as percent relative fluorescent units (RFU) of the untreated WT mean control value ± SEM. N = CMs obtained from four different hearts. *p < 0.05, **p < 0.01, ***p < 0.001 compared to mean control value. (c) Summarized nitrite assay data depicting the effects of AITC (0–300 µM) and vehicle (Veh; EtOH) on nitrite production in CMs obtained from WT and TRPA1^-/-^ hearts. Absorbance was recorded at 540 nm. Data are expressed as the amount of nitrite produced per mg of sample protein. N = CMs obtained from four different hearts and data collected from 10 assays. *p < 0.05, **p < 0.01, ***p < 0.001 compared to vehicle-treated control value within the respective CM cohorts.

WT CMs were treated with AITC (100 μM) for various time durations (0, 2, 5, 10 min), lysed and prepared for immunoblot assessment. Utilizing antibodies generated to recognize phosphorylated AktpS^473^ and eNOSpS^1177^, we determined that AITC elicits AktpS^473^ and eNOSpS^1177^ beginning at 2 min but most prominently at 5 min in WT CMs (183 ± 6.7% and 157 ± 8.0% of mean control value, respectively; Figure 1(a)). Summarized data is depicted in Figure 1A’. WT and TRPA1^-/-^ CMs were also loaded with an NO detection reagent and prepared for fluorescence microscopy in order to examine whether eNOS phosphorylation occurs concomitantly with NO production. CMs isolated from WT and TRPA1^-/-^ mice were subjected to AITC (100 μM) treatment for various time durations to examine whether NO production by TRPA1 stimulation occurs in a time-dependent manner. Fluorescence microscopy demonstrated that AITC induces NO production at 2 min, but most intensely at 5 min in a single WT CM (142 ± 4.1%) – an effect that was not observed in CMs obtained from TRPA1^-/-^ mice (103 ± 5.3%; Figure 1(b)). Summarized data is depicted in Figure 1B’.

### TRPA1 activation with AITC stimulates NO production in a concentration-dependent manner in CMs

In order to determine if TRPA1 stimulation elicits NO production, WT and TRPA1^-/-^ CMs were subjected to a nitrite assay protocol in order to examine the extent to which nitrite production (a more stable breakdown product of nitric oxide) is altered by varying dosages of AITC. CMs loaded with nitrite detection reagents were subjected to AITC (0–300 μM) treatments and nitrite production was quantified. AITC induced nitrite production in WT CMs in a concentration-dependent manner (Figure 1(c)). The AITC-induced nitrite production was not observed in CMs obtained from TRPA1^-/-^ mice.

### TRPA1-induced increases in CM [Ca^2+^]_i_ and contractile function occur independently of Akt and eNOS

**Figure 2.**
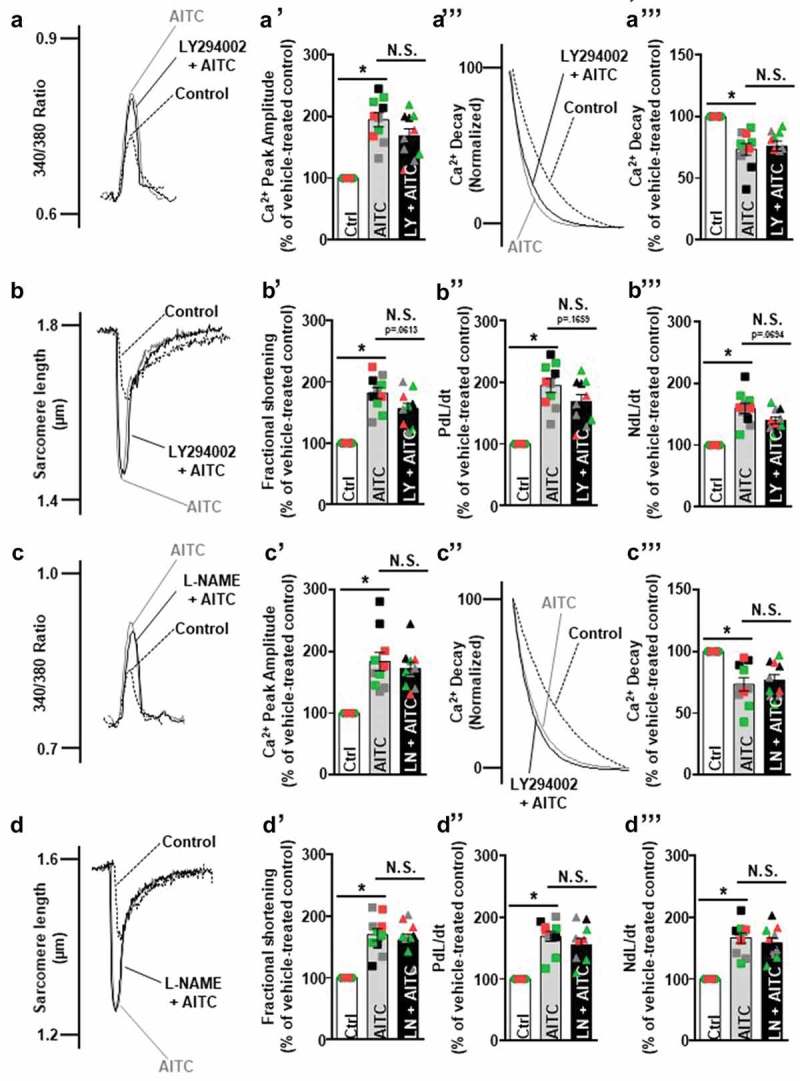
TRPA1-induced increases in CM contractile function occur independently of Akt and eNOS. Overlays illustrating the effects of AITC (100 µM) in the presence and absence of Akt inhibitor, 2-Morpholin-4-yl-8-phenyychromen-4-one (LY294002; 10 µM), or NOS inhibitor, L-NG-nitroarginine methyl ester (L-NAME; 100 µM), on CM calcium dynamics and contractile function. (A-A’, C-C’) AITC increases peak [Ca^2+^]_i_, (B-B’, D-D’) fractional shortening, (B’’, D’’) maximum velocity of cell shortening and (B’’’, D’’’) maximum velocity of cell relengthening while (A’’-A’’’, C’’-C’’’) accelerating the rate of [Ca^2+^]_i_ decay (each peak normalized to 100% of peak Ca^2+^ amplitude) in CMs. (a,b) Addition of LY294002 or (c,d) L-NAME did not alter any of these parameters to a significant extent. Summarized data are expressed as a percent of steady-state baseline control (Ctrl) value set at 100%. Changes in [Ca^2+^]_i_ are measured as the change in the 340/380 ratio. Changes in timing were measured in milliseconds. Individual traces were smoothed using the Savitzky-Golay filter to increase the signal-to-noise ratio. Changes in fractional shortening were measured as percent of sarcomere length. Changes in velocity were measured in micrometers/sec. N = CMs obtained from ten CMs collected from four different hearts. CMs from the same heart are indicated by color. N.S., not significant. *p < 0.001 compared to control value.

WT CMs were electrically paced using a field stimulator and treated with AITC (100 µM) in the presence or absence of Akt inhibitor, LY294002 (10 μM), or NOS inhibitor, L-NAME (100 μM), and assessed for alterations in calcium cycling dynamics and contractile function. Figure 2(a,c) depict representative overlaid [Ca^2+^]_i_ traces, where Figure 2(b,d) represent overlaid shortening traces of a single CM that was untreated (control) before AITC administration and subsequent LY294002 or L-NAME/AITC co-treatment. Treatment with AITC accelerates the rate of [Ca^2+^]_I_ decay (73.4 ± 4.7%; **Fig. 2A’’-A’’’** and 73.4 ± 5.4%; **Fig. 2C’’-C’’’**) and increases peak [Ca^2+^]_I_ (189 ± 9.2%; Figure 2(a)**-A’** and 184 ± 15%; Figure 2(c)**-C’**), fractional shortening (182 ± 9.0%; Figure 2(b)**-B’** and 170 ± 9.6%; Figure 2(d)**-D’**), maximum velocity of shortening (195 ± 11%; **Fig. 2B’’** and 169 ± 8.2%; **Fig. 2D’’**) and maximum velocity of relengthening (160 ± 8.3%; **Fig. 2B’’’** and 167 ± 8.3%; **Fig. 2D’’’**), as we previously demonstrated [9]. Addition of LY294002 or L-NAME did not significantly alter any of these parameters (Figure 2(a–d)).

### TRPA1^-/-^ CMs exhibit lower baseline expression levels of phosphorylated enosps^1177^ in comparison to WT

**Figure 3.**
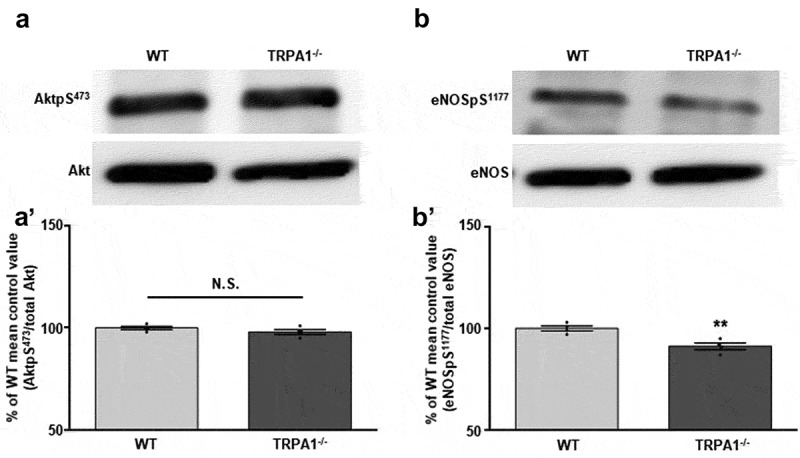
TRPA1^-/-^ CMs exhibit lower baseline expression levels of eNOSpS^1177^, but not AktpS^473^, compared to WT. Representative immunoblots depicting the basal expression levels of (a) AktpS^473^ and (B) eNOSpS^1177^ in WT and TRPA1^-/-^ CMs. Total Akt and eNOS were probed as loading controls. (A’) Summarized data for Figure 3(a) and (B’) Figure 3(b). Data are expressed as a percent of the WT control mean value ± SEM. *p < 0.05, **p < 0.01, ***p < 0.001 compared to WT control value.

CMs obtained from WT and TRPA1^-/-^ mice were prepared for immunoblotting in order to determine baseline expression levels of phosphorylated Akt and eNOS. Immunoblot assessment indicated that while no immunodetectable differences were observed in basal AktpS^473^ (98.0 ± 1.2% of control; Figure 3(a)), eNOSpS^1177^ expression levels were significantly lower in TRPA1^-/-^ CMs when compared to those obtained from WT mice (91.3 ± 1.7%; Figure 3(b)). Total Akt and eNOS protein levels were not significantly different between the two strains of mice. Summarized data are depicted in Figures 3A’ and 3B’.

### AITC induces eNOS phosphorylation at serine 1177 through an Akt-dependent, TRPA1-mediated signal transduction pathway in CMs

**Figure 4.**
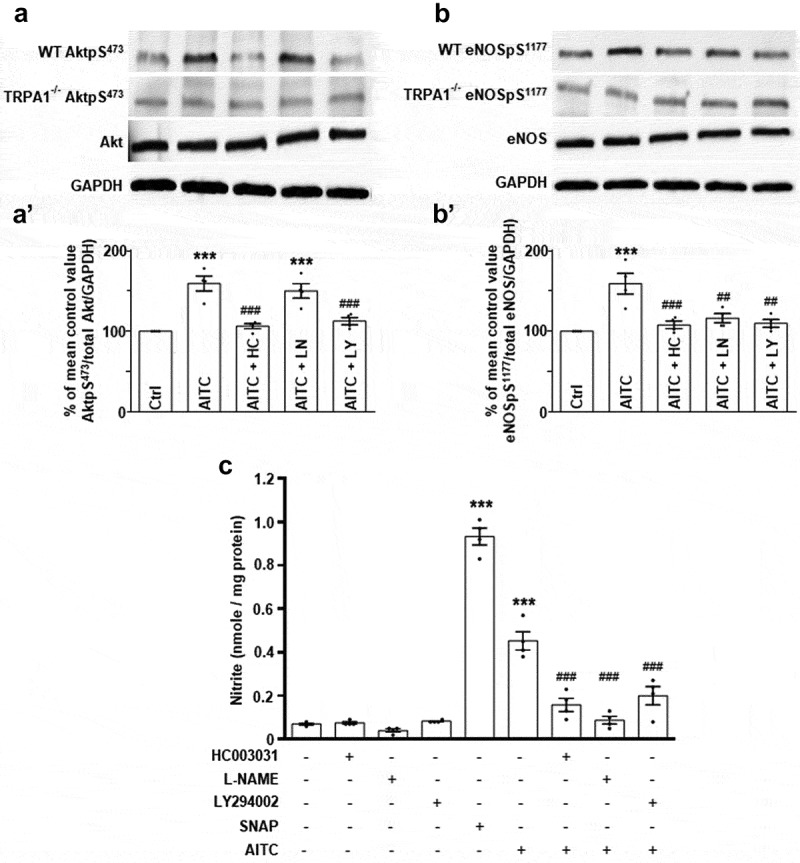
AITC induces nitrite production through a TRPA1/Akt/eNOS-mediated mechanism in CMs. (a) Representative immunoblots depicting the effect of AITC (100 µM) in the presence or absence of the TRPA1 antagonist, HC-030031 (1 µM), L-NAME (100 µM) or LY294002 (10 µM) on AktpS^473^ and (b) eNOSpS^1177^ expression levels in CMs obtained from WT or TRPA1^-/-^ murine hearts. Total Akt, eNOS, and GAPDH were probed as loading controls. (A’) Summarized data for Figure 4(a) and (B’) Figure 4(b). Immunoblot data are expressed as a percent of the untreated control mean value (phosphorylated protein/total amount of respective protein/GAPDH) ± SEM. N = CMs obtained from four different hearts. (c) Summarized nitrite assay data depicting the effects of AITC in the presence or absence of HC-030031, L-NAME or LY294002 on CMs obtained from WT hearts. NO donor, S-Nitroso-N-Acetyl-D, L-Penicillamine (SNAP; 10 µM), was utilized as a positive control. Absorbance was recorded at 540 nm and data are expressed as the amount of nitrite produced per mg of sample protein. N = CMs obtained from four different hearts and data collected from 10 assays. *p < 0.05, **p < 0.01, ***p < 0.001 compared to mean control value. #p < 0.05, ##p < 0.01, ###p < 0.001 compared to AITC-treated value.

In order to elicit the signal transduction pathway induced by TRPA1 stimulation, WT and TRPA1^-/-^ CMs were untreated or pretreated with HC-030031 (1 μM), L-NAME (100 μM) or LY294002 (10 μM) prior to AITC (100 μM) administration. Subsequently, the samples were prepared for immunoblot analysis utilizing antibodies recognizing AktpS^473^ and eNOSpS^1177^. Immunoblot assessment demonstrates that AITC induced elevated phosphorylation levels of AktpS^473^ (157 ± 8.3% of control; Figure 4(a)) and eNOSpS^1177^ (161 ± 11% of control; Figure 4(b)) in WT CMs when compared to the vehicle-treated control. The AITC-induced increase in AktpS^473^ returned to near baseline levels when pretreated with HC-030031 (108 ± 3.4%) and LY294002 (113 ± 4.7%); however, pretreatment with L-NAME yielded results statistically similar to those obtained with AITC treatment alone (149 ± 9.1%; Figure 4(a)). AITC-induced increases in eNOSpS^1177^ returned to near baseline levels when CMs were pretreated with HC030031 (110 ± 3.7%), L-NAME (120 ± 5.2%) and LY294002 (111 ± 4.4%; Figure 4(b)). Parallel experiments were conducted in CMs obtained from TRPA1^-/-^ mice. Immunoblot analysis of TRPA1^-/-^ CMs exposed to AITC in the presence and absence of HC030031, L-NAME or LY294002 indicates that none of the treatment groups had any immunodetectable effects on AktpS^473^ or eNOSpS^1177^ expression levels. AktpS^473^ and eNOSpS^1177^ were normalized to total Akt and eNOS protein levels detected in the CM lysates, respectively. GAPDH was probed as the loading control. Summarized data are depicted in Figures 4A’ and 4B’.

### TRPA1 stimulation elicits nitrite production in CMs through an Akt/eNOS-mediated mechanism

In order to elucidate the mechanism by which AITC stimulates nitrite production, HC-030031, L-NAME and LY294002 were utilized as pretreatments prior to TRPA1 agonist administration. SNAP (NO donor; 0.93 ± 0.04 nmol/mg) and AITC (0.45 ± 0.04 nmol/mg) stimulated significant increases in nitrite production in WT CMs, whereas pretreatments with HC-030031 (0.16 ± 0.03 nmol/mg), L-NAME (0.09 ± 0.02 nmol/mg) and LY294002 (0.20 ± 0.04 nmol/mg) prior to AITC administration returned nitrite production to near basal levels (Figure 4(c)). Treatments with HC030031 (0.08 ± 0.01 nmol/mg), L-NAME (0.04 ± 0.01 nmol/mg) or LY294002 (0.08 ± 0.0 nmol/mg) alone had no significant effects on nitrite production in CMs.

### Activation of TRPA1 in CMs attenuates ischemia-induced cell death through an eNOS-mediated mechanism

**Figure 5.**
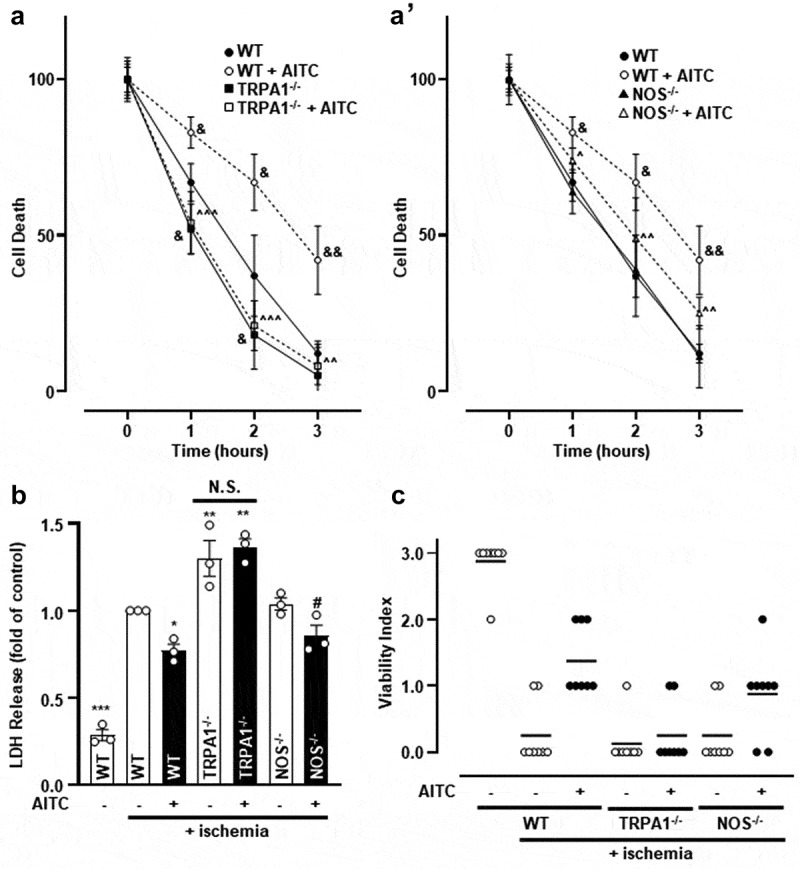
The presence and activation of TRPA1 protects CMs from ischemia-induced death. (a) Summarized data depicting the extent to which WT, TRPA1^-/-^ and (A’) NOS^-/-^ CMs undergo ischemia-induced cell death in the presence or absence of AITC (100 µM) over the course of 3 h. Data are expressed as a percent of pro-caspase 3 (pre-apoptotic marker) to cleaved caspase (post-apoptotic marker) ratio at 0 h. GAPDH was probed as the loading control. (b) Summarized data demonstrating lactate dehygrogenase (LDH) release in WT, TRPA1^-/-^ and NOS^-/-^ CMs treated with or without AITC for 3 h. WT CMs exposed to ischemia-mimetic buffer were set as the control value at one and remaining data are expressed as a fold of control. (c) Viability index scored by gross assessment of CMs in the presence or absence of ischemia-mimetic buffer-treated with or without AITC. Mean score is represented as a line. (&) indicates significance compared to untreated WT CMs at a corresponding time point, (^) compared to WT CMs treated with AITC at a corresponding time point, (*) indicates significance compared to ischemia-treated WT CMs, (#) indicates significant compared to ischemia-treated NOS^-/-^ CMs. One symbol (p < 0.05), two symbols (p < 0.01), three symbols (p < 0.001). N = CMs obtained from (a) four, (b) three and (c) eight hearts.

A previous study demonstrated that TRPA1 activation elicits cardioprotection *in vivo* [10]. In order to elucidate the cellular signal transduction pathway downstream of TRPA1-mediated protection, we employed an *in vitro* protocol utilizing a buffer designed to mimic the extracellular milieu observed in severe hypoxia and ischemia. CMs obtained from WT, TRPA1^-/-^ or NOS^-/-^ mice were cultured in an ischemia-mimetic buffer in the presence of either AITC (100 µM) or vehicle and assessed for cell death over the course of 3 h. Initially, a control experiment was conducted with CMs obtained from WT mice cultured in the ischemia-mimetic buffer to determine the rate at which cell death occurs in our *in vitro* preparation. AITC attenuated ischemia-induced cell death in CMs obtained from WT mice in as early as the first hour, but not in TRPA1^-/-^ (Figure 5(a)) or NOS^-/-^ murine CMs (**Fig. 5A’**). Moreover, the rate at which TRPA1^-/-^ CMs undergo cell death was accelerated compared to WT CMs. This effect was absent in CMs obtained from NOS^-/-^ hearts. Assays designed to measure lactate dehydrogenase (LDH) release were conducted to determine the extent to which WT, TRPA1^-/-^, or NOS^-/-^ CMs release LDH in response to ischemic insult in the presence or absence of AITC. WT and NOS^-/-^ CMs treated with AITC demonstrated lower LDH release levels compared to their vehicle-treated counterparts (Figure 5(b)). CMs obtained from TRPA1^-/-^ hearts exhibited increased LDH release compared to WT controls. Finally, a scoring index was created in order to assess the viability of cells exposed to ischemic insult for 3 h (**Supp. Table 1**). WT and NOS^-/-^ CMs treated with AITC demonstrated overall preserved cell viability compared to their vehicle-treated counterparts (Figure 5(c)).

## Discussion

The current study is among the first to elucidate signal transduction pathways elicited via TRPA1 stimulation in cardiac muscle tissue. We previously identified the functional expression and localization of TRPA1 in the cardiac muscle where activation of the ion channel leads to enhanced [Ca^2+^]_i_ and contractile function through a CaMKII-dependent process [8,9]. Preliminary biochemical evidence obtained from our lab suggested that TRPA1 activation may precede Akt and eNOS phosphorylation in CMs. Taken together with previous evidence in the literature indicating a role for TRPA1, Akt and nitric oxide in modulating cardiac contractility [9,11,25], we hypothesized that Akt and eNOS are necessary for TRPA1-induced increases in CM contractile function. Our key findings include: 1) TRPA1 stimulation elicits NO production and post-translational modifications of Akt at serine 473 and eNOS at serine 1177 (each of which are indicators of protein activation [26,27]), 2) TRPA1-induced increases in CM [Ca^2+^]_i_ and contractile function occur independently of Akt and eNOS activation and 3) the presence and activation of TRPA1 protects against ischemia-induced cardiomyocyte cell death.

### TRPA1 stimulation elicits Akt and eNOS phosphorylation in CMs

Our first objective was to determine whether TRPA1 agonist (AITC) treatment leads to increased AktpS^473^ or eNOSpS^1177^ phosphorylation. Our current results demonstrate that AITC induces AktpS^473^ and eNOSpS^1177^ phosphorylation by 2 min, but most significantly at 5 min following treatment. These data are consistent with previous findings in our laboratory that show TRPA1 induces phosphorylation of nitric oxide synthases in F-11 cells [13]; however, the reporting of this effect occurring in CMs is a novel finding. As eNOSpS^1177^ is an indicator of activation rendering it catalytically viable, we subsequently assessed the extent to which TRPA1 stimulation affects NO production in CMs.

Notably, the possibility exists of AITC activating additional signaling pathways capable of producing NO when administered at high concentrations. In fact, it has previously been shown that AITC can affect other Ca^2+^ channels (e.g. TRPV1), but this only occurs when concentrations of AITC reach the millimolar range [28–30]. In the current study, we found that AITC treatment leads to increases in NO production 5 min following treatment and elevated nitrite production in a concentration- and TRPA1-dependent manner in CMs. Previous studies from our lab have demonstrated the specificity of AITC for TRPA1 through concentrations up to 500 µM [31]; however, the current data suggest that specificity of AITC-induced, TRPA1-mediated nitrite production is lost once concentrations of AITC reach 300 µM. As such, all subsequent experiments were conducted using AITC at 100 μM.

In order to begin delineating the precise mechanism by which AITC elicits AktpS^473^ in CMs, we employed an *in vitro* approach utilizing pharmacological inhibitors. In the current studies, the AITC-induced increase in AktpS^473^ expression levels was markedly attenuated when CMs were pretreated with HC-030031 and LY294002, but not when pretreated with L-NAME. Furthermore, the agonist-induced increases in nitrite production are substantially attenuated in the presence of HC030031 and LY294002, while being completely eliminated in the presence of L-NAME. This data suggests that AITC-induced Akt activation is TRPA1-dependent and occurs upstream of eNOS activation in CMs. Furthermore, activation of Akt may elude to the precise functional relevance of this TRPA1-mediated mechanism in cardiac tissue. The relative promiscuity of Akt involvement in multiple cellular signal transduction pathways indicates its ability to modulate several cellular processes and makes it an attractive target for therapeutic intervention [23]. Several studies have demonstrated the role of Akt in physiological events occurring throughout the myocardium. Precisely, phosphorylation of AktpS^473^ has been implicated as a major player in numerous myocardial cellular processes. Akt has been demonstrated to be involved in physiological events such as enlarging cell size [25], exerting positive inotropic effects on cardiomyocyte contractility [25] through modulation of LTCC calcium influx [32], increasing sarcoplasmic reticulum Ca^2+^-ATPase levels [33] and augmenting phospholamban phosphorylation [34]. Surprisingly, the current findings indicate that TRPA1-induced increases in CM contractility occur independently of Akt even though TRPA1 stimulation elicits increases in AktpS^473^ (Figure 2). This suggests that this activation pathway may be occurring in parallel with additional signaling cascades involving CaMKII [9] that modulate the effects of TRPA1 on contractility. These effects are likely due to CaMKII-mediated changes in phospholamban, SERCA and/or troponin I activity which are known to result in positive inotropic and lusitropic effects, as we noted in our previous publication [9].

Numerous studies have demonstrated the role of Akt in eNOSpS^1177^ phosphorylation, both at the basal and stimulated levels [35,36], and many of these investigations discuss the importance of the Akt-eNOS signaling cascade in vascular tissue. In the current studies, HC-030031, L-NAME, and LY294002 substantially attenuated the agonist-induced increase in eNOSpS^1177^. Parallel experiments performed in TRPA1^-/-^ CMs demonstrated complementary results, in which treatments with AITC did not elicit any significant alterations in phosphorylated eNOSpS^1177^ expression levels. The current findings are consistent with previous studies demonstrating TRPA1-dependent NOS isoform alterations in a variety of cell types including sensory neurons located in the periphery [37,38]. Increases in eNOSpS^1177^ phosphorylation are also observed during cardiac muscle stretch, which directly mediates an alteration in excitation-contraction coupling [12,39]. Additionally, recent studies have demonstrated that phosphodiesterase type 1 inhibitors block hydrolysis of the NO downstream effector, cyclic GMP (cGMP), effectively increasing inotropy, lusitropy and vasodilation in dogs and rabbits [40]; however, an earlier investigation demonstrated that the catalytic enzyme of cGMP formation, soluble guanylyl cyclase, is not required for nitroxyl-induced positive inotropic effects in mice [41]. Given the conflicting results reported in the literature, we investigated the extent to which eNOS is involved in TRPA1-induced increases in CM contractility. The current findings suggest the enhanced CM contractile function occurring as a result of TRPA1 activation occurs independently of eNOS, further validating a potential divergence in TRPA1-mediated signaling pathways where TRPA1-induced CaMKII stimulation may affect downstream mediators of contractility.

The average diastolic sarcomere length (SL) observed in this study (1.76 ± 0.2 µm) is similar to those found in other studies using freshly isolated or cardioplegically arrested cells with a range of 1.71 ± 0.1 µm to 1.93 ± 0.1 µm [42–48]. The variation in these values is likely due to the different methods used to assess SL (i.e. optical, x-ray diffraction, histological). However, the values obtained from these studies, as well as our own, are shorter than values obtained in Langendorff perfused hearts labeled with the membrane dye di-4-ANNEPS using remote focusing microscopy [49] where the absolute value of diastolic SL was 2.09 ± 0.16 µm. There are several factors that may contribute to these differences. Isolation of single cells or cardiac muscle slices for in vitro studies may alter a variety of complex architectural interactions that occur in the intact heart leading to a reduction in a passive strain which could ultimately affect cell length and reduce SL – a potential state of CM hypercontracture. Consequently, SL in isolated cell preparations will be shorter than that observed in the intact heart. Along the same lines, hypercontracture could result from abnormally high diastolic Ca^2+^ depending on the cell viability and/or buffer composition resulting in an unphysiological baseline or starting point. On the other hand, tissue extraction approaches typically will result in the release of pressure from the pericardium ultimately culminating in tissue edema that would be associated with a corresponding increase in SL, as noted in one elegant study by Botcherby et al., 2013 [49]. In this case, SL in Langendorff perfused hearts will be greater than that observed in the intact animal. Although isolated cells and perfused heart preparations have been used for decades to assess cardiac architecture and function, the development of minimally invasive imaging approaches will be required to facilitate comparison between in vitro and in situ experimental approaches of assessing cardiac muscle architecture and its relationship to cardiac function.

We observed several indications of diastolic calcium release in our isolated CM preparations, as shown during the post-diastolic, pre-systolic relaxation period of Figure 2(a,c). Although these occurrences were rare, it is worthy to note that this could be due to mechanical interference with cellular structures causing local Ca^2+^ sparks, as previously suggested [50,51]. Another attributable cause could be an unstable preparation of isolated CMs; however, our experiments regularly consist of electrically stimulated CM pacing over long durations of time (up to 20 min) after which point the cells retain a rod-shaped morphology and clear striations, as well as a lack of membrane blebbing. This suggests that cellular integrity and viability of the CM preparation are stable and well preserved throughout the duration of the experimental protocol.

Despite the controversial nature of the role of NO production with regards to CM contractility [12], the current data suggests AITC (100 µM) increases NO production and subsequent nitrite formation. The extent to which TRPA1 regulates basal NO production may be of future interest; however, since HC030031 alone only slightly diminishes NO production, TRPA1 does not appear to regulate basal NO production to a significant degree in CMs. Indeed, CMs obtained from TRPA1^-/-^ mice did not exhibit significant alterations in basal NO or nitrite production when compared to WT.

The current results also suggest that phosphorylated eNOSpS^1177^ expression levels in CMs obtained from TRPA1^-/-^ mice are significantly lower than those from WT mice. The diminished eNOSpS^1177^ expression levels confer a potential phenotypic alteration of NO handling in cardiac tissue obtained from TRPA1^-/-^ mice. Although the diminished levels of eNOSpS^1177^ observed in TRPA1^-/-^ mice should theoretically modify basal cardiac function, one previous study found no significant differences in basal heart rate, size, function or blood pressure between TRPA1 null and WT mice [52]. However, the cardioprotection afforded by eNOSpS^1177^ and moderate NO levels may confer susceptibility of TRPA1^-/-^ mice to exacerbated injury following ischemic insult. Moreover, endogenous NO has been implicated in suppressing inflammation and improving myocardial protection following ischemia/reperfusion injury through a mechanism by which it modulates the opening and closing of the mitochondrial permeability transition pore [53–55]. On the other hand, exogenously applied NO has been shown to alter mitochondrial-mediated intracellular machinery in response to oxidative stress by which it induces the production of superoxide anions and possibly peroxynitrite [56]. In this sense, NO application has the potential to exacerbate pathological conditions by which it induces the production of harmful superoxide and peroxynitrite suggesting a bimodal effect of the second messenger in response to ischemic insult. Given the extensive uncertainty afforded by the bimodal regulatory nature of NO in cardiac tissue, we subsequently assessed the extent to which TRPA1-induced NO production is involved in ischemia-induced CM cell death.

### AITC-mediated NOS activation partially protects CMs from ischemia-induced cell death

Extensive evidence has demonstrated prominent roles for Akt and eNOS in modulating pathophysiological conditions in the heart. Akt has been implicated in protection from ischemia-reperfusion injury in isolated adult rat cardiomyocytes [57,58], as well as promoting cell survival through manipulation of anti-apoptotic proteins and the mitochondrial-associated apoptotic machinery [22]. Furthermore, eNOSpS^1177^ has been implicated in serving a role in cardioprotection following ischemic insult in the heart [13,15] and eNOSpS^1177^ phosphorylation has been demonstrated to be protective against apoptosis *in vitro* [59]. Therefore, diminished expression levels of eNOSpS^1177^ may render TRPA1^-/-^ CMs more susceptible to exacerbated injury. As such, we aimed to determine whether the presence or activation of TRPA1 protects against ischemia-induced cell death. The current findings suggest CMs obtained from TRPA1^-/-^ mice undergo cell death at an accelerated rate and exhibit increased LDH release compared to WT CMs. Furthermore, WT CMs treated with AITC demonstrate attenuated cell death and LDH release when compared to the vehicle-treated control cells; this effect was absent in CMs obtained from TRPA1^-/-^ and NOS^-/-^ mice suggesting that the protective effects afforded by AITC rely upon the presence of TRPA1 as well as eNOS. The existence of this signaling pathway in CMs is consistent with our previous findings demonstrating that TRPA1 stimulation restores TRPV1 sensitivity through an eNOS-mediated pathway in dorsal root ganglion neurons [60].

### Summary and conclusions

Overall, the current findings are consistent with previous findings indicating that TRPA1 stimulation elicits NOS isoform activation and NO production; however, the identification of a TRPA1/Akt/eNOS/NO mechanism in freshly isolated CMs is novel in nature. The current data suggests TRPA1-mediated Akt/eNOS activation and subsequent NO production elicit protective effects in isolated murine CMs. Additionally, this protective axis does not appear to be involved in TRPA1-induced activation of CaMKII and consequential increases in CM [Ca^2+^]_i_ and contractility. Therefore, it’s likely that effects on CM [Ca^2+^]_i_ and contractility elicited by TRPA1 stimulation occur through direct alterations of LTCCs, phospholamban and/or troponin I by CaMKII whereas a parallel pathway promoting protection is carried out by Akt, eNOS and NO. The polymodal activation mechanisms and intricate regulatory functions of Akt, eNOS and NO suggest a notable complexity of cellular signal transduction pathways to which they are involved. Furthering our understanding of the mechanisms initiated via TRPA1 stimulation in cardiac tissue could provide important fundamental insight into the role of TRPA1 ion channels in the physiology and pathophysiology of cardiac muscle.

## Materials and methods

### Animal models

Four-month-old male WT and TRPA1^-/-^ mice (Jackson Laboratories) were utilized and maintained in accordance with the *Guide for the Care and Use of Laboratory Animals* (NIH). Kent State University (Kent, OH) and Northeast Ohio Medical University (NEOMED) animal care facilities housed all animals, which is accredited by the American Association for Accreditation of Laboratory Animal Care.

### CM isolation

Murine hearts were excised, prepared for aortic cannulation and transferred to a Langendorff apparatus for CM isolation, as previously described [8]. In brief, hearts underwent retrograde perfusion at 37°C and pH 7.4 with a modified Krebs-Henseleit buffer (in mM: 120.4 NaCl, 4.8 KCl, 0.6 KH_2_PO_4_, 0.6 Na_2_HPO_4_, 1.2 MgSO_4_-7H_s_O, 10 Na-HEPES, 4.6 NaHCO_3_, 30 taurine, 10 BDM, and 5.5 glucose). The Krebs-Henseleit buffer was sterile-filtered and paced at a rate of 4 mL/min. After perfusion for 4 min, the digestion buffer containing collagenase type II (309 U/mg, Worthington Biochemical) perfused the heart for an additional 5–7 min until the heart became spongy. The left ventricles were removed, minced, and triturated in Krebs-Henseleit buffer containing fetal bovine serum. The resulting cellular digest was washed and resuspended at 23°C in HEPES-buffered saline (in mM: 118 NaCl, 4.8 KCl, 0.6 KH_2_PO_4_, 4.6 NaHCO_3_, 0.6 NaH_2_PO_4_, 5.5 glucose, pH 7.4). CM yield was consistently ~80–90%.

### Preparation of cell lysates for immunoblot analysis

Immunoblot analysis was performed as previously described [61]. CMs were homogenized and protein concentration was subsequently assessed using the Bradford method [62]. All samples were adjusted to ~2 mg/mL protein concentration. Samples containing 50 µg of protein lysates were boiled and subjected to SDS-PAGE on 4–15% precast polyacrylamide gels (Bio-Rad) through the use of a minigel apparatus. After running, gels were then transferred to nitrocellulose membranes. Nonspecific binding was blocked with 1% BSA solution in Tris-buffered saline solution (0.1% [vol/vol] Tween-20 in 20 mM Tris base, 137 mM NaCl, pH 7.6) for 1 h at room temperature. Polyclonal antibodies recognizing phosphorylated Akt at serine 473, total Akt, phospho-eNOS serine 1177 (Cell Signaling), total eNOS (Affinity Bioreagents) and a monoclonal antibody recognizing total GAPDH (Millipore) were diluted 1:1000 in Tris-buffered saline containing 1% BSA and incubated at 4°C overnight. After washing, membranes were incubated for 1 h at room temperature with horseradish-peroxidase-linked secondary antibody (goat anti-rabbit and goat anti-mouse) diluted 1:5000 in Tris-buffered saline with 1% BSA. Enhanced chemiluminescence was used for antibody detection utilizing an ImageQuant LAS 4000 Mini (General Electric). Immunoreactivity was assessed by scanning densitometry and analyzed using ImageJ software (NIH).

### Measurement of NO production

NO production in CMs isolated from WT and TRPA1^-/-^ mice was assessed using a ROS-ID NO detection kit (Enzo Life Sciences) to determine the time frame of NO production following treatment. The protocol and dilution of reagents were conducted in accordance with the kit instructions. Once CMs were isolated, the cells were incubated with the cell-permeable fluorescent probe used for the detection of NO for 2 h at 37°C in light-sensitive conditions. CMs were then rinsed with wash buffer, subjected to AITC (100 µM) treatment and subsequently prepared for fluorescence/confocal microscopy using an Olympus IX-70, FV5 PSU (Olympus America) and a Cyanine 5 filter (650/670 nm). To standardize selection and fluorescence of CMs, the exact parameters used for control samples (gain = 500 V, 10% full power) were utilized in all treatment group measurements. Results were assessed via ImageJ and data is expressed as percent relative fluorescent units (%RFU; the percentage of untreated control CMs).

### Detection of nitrite production

NO has an extremely short half-life and quickly metabolizes to nitrate and nitrite. Therefore, we utilized a colorimetric nitrite assay kit (Sigma–Aldrich) to measure nitrite levels, which was used to indicate NO production in a more stable format. All nitrite assay experiments were performed per the manufacturer’s protocol. In brief, an equal volume of detection reagents were added to 96-well plates with Hank’s balanced salt solution (in mM: 120 NaCl, 5 KCl, 1 MgCl_2_, 5.5 glucose, 10 HEPES, 1.23 CaCl_2_, pH 7.4) containing CMs. Azo dye production was quantified by absorbance at 540 nm in room temperature using an Emax precision microplate reader (Molecular Devices). Nitrate production was quantified utilizing standardized reagents which produced a standard curve against which we plotted our absorbance recording. Results were normalized to each other by lysing the individual samples and subsequential protein concentration assessment. Results are reported as the amount of nitrite produced per mg of sample protein.

### Simultaneous measurement of [Ca^2+^]_i_ and shortening

Simultaneous measurement of [Ca2+]i and contractile function was performed in individual freshly isolated CMs as previously described by our laboratory [9]. CMs were incubated at room temperature for 30 min with fura-2 acetoxy methylester (fura-2/AM; 2 µM) in HEPES-buffered saline (in mM: 118 NaCl, 4.8 KCl, 1.23 CaCl2, 0.8 MgSO4-7H2O, 0.6 KH2PO4, 4.6 NaHCO3, 0.6 NaH2PO4, 5.5 glucose, pH 7.4). Coverslips containing the fura-2–70-loaded CMs were then mounted on the stage of an Olympus IX-71 inverted fluorescence microscope (Olympus America). CMs were superfused continuously with HEPES-buffered saline at a flow rate of 2 mL/min and compounds were introduced in a dose-dependent manner. Sarcomere shortening and [Ca^2+^]_i_ measurements were simultaneously recorded on individual cells using the fluorescence imaging system and Easy Ratio Pro software (Photon Technology International) equipped with a multiwavelength spectrofluorometer (Deltascan RFK6002) and a QuantEM 512SC electron multiplying camera (Photometrics). Images and real-time calcium tracing data were acquired using an alternating excitation wavelength protocol (340, 380 nm/20 Hz) and an emission wavelength of 510 nm. Background fluorescence was automatically corrected for the experiments using Easy Ratio Pro. The ratio of the two intensities was used to measure changes in [Ca^2+^]_i_ due to the fact that calibration of the system relies upon a number of assumptions. Hardware and software for data acquisition and analysis were generously provided by Horiba Scientific (Edison, NJ).

### Analysis of [Ca^2+^]_i_ and shortening data

The following variables were calculated for each individual contraction: sarcomere length (µm), fractional shortening (% of sarcomere length change during shortening), maximum velocity of cell shortening and relengthening (µm/sec), peak [Ca^2+^]_i_ (340/380 ratio) and [Ca^2+^]_i_ decay to baseline (msec). Variables from 10 contractions were averaged to obtain mean values at baseline and in response to the intervention. Averaging the variables over time minimizes beat-to-beat variation. The summarized shortening raw data are expressed as % change in sarcomere length (fractional shortening) and mm/sec (velocity of shortening/relengthening). The summarized [Ca^2+^]_i_ raw data are expressed in msec. Individual [Ca^2+^]_i_ transient traces were smoothed using the SavitzkyGolay filter to increase the signal-to-noise ratio and enhance the clarity of the figure to highlight changes in timing parameters.

### In vitro ischemia-mimetic protocol

Freshly isolated CMs obtained from wild-type and TRPA1^-/-^ mice were incubated in a buffer designed to simulate the ischemic environment during MI (Esumi et al., 1991; Zhao et al., 2014) and prepared for immunoblot, lactate dehydrogenase release assays or viability assessment. Immunoblot data were calculated as the ratio of pro-caspase 3 (pre-apoptotic marker) to cleaved caspase (post-apoptotic marker) and are expressed as a percent of the 0-h control. LDH release assays were performed according to manufacturer instructions. In brief, samples were homogenized, centrifuged, and LDH activity was measured in the supernatant and incubation media. Results are expressed as a fold of the WT vehicle-treated CM control. Viability was assessed by a pre-determined index score system listed in the supplemental data. Average score value for each treatment group is represented as a solid black line.

### Statistical analysis

Results obtained from each animal were averaged so that all animals were weighted equally. Student’s t-test, one- and two-way ANOVA as well as Bonferroni *post hoc* test were used where appropriate. Statistical significance is indicated as p < 0.05 (one symbol), p < 0.01 (two symbols) and p < 0.001 (three symbols). All results are expressed as mean ± SEM. Statistical analysis was conducted using Prism 6.0 (GraphPad software).
