# Supplementary material for: Stimulation of TRPA1 attenuates ischemia-induced cardiomyocyte cell death through an eNOS-mediated mechanism
Source: Channels (Austin). 2019 Jun 4;13(1):192–206. doi: 10.1080/19336950.2019.1623591 (PMC6557600; doi:10.1080/19336950.2019.1623591)
Supplement: Supplemental Material [file kchl-13-01-1623591-s001.zip › Supplemental caption.docx]

**Supplemental Table 1: Viability Index Scores.** CMs were scored 0-3 based on gross assessment of viability parameters such as rod-shaped morphology, clear striations and no membrane blebbing.

**Supplemental Figure 2: LDH release is markedly reduced in WT and NOS^-/-^ CMs treated with AITC over the course of three hours.** Summarized data demonstrating lactate dehygrogenase (LDH) release in WT, TRPA1^-/-^ and NOS^-/-^ CMs treated with or without AITC at zero, one, two or three hours. WT CMs exposed to ischemia-mimetic buffer for three hours were set as the control value at one and remaining data are expressed as a fold of control. N = CMs obtained from three hearts.
