# Supplementary figures and images for: Stimulation of TRPA1 attenuates ischemia-induced cardiomyocyte cell death through an eNOS-mediated mechanism
Source: Channels (Austin). 2019 Jun 4;13(1):192–206. doi: 10.1080/19336950.2019.1623591 (PMC6557600; doi:10.1080/19336950.2019.1623591)

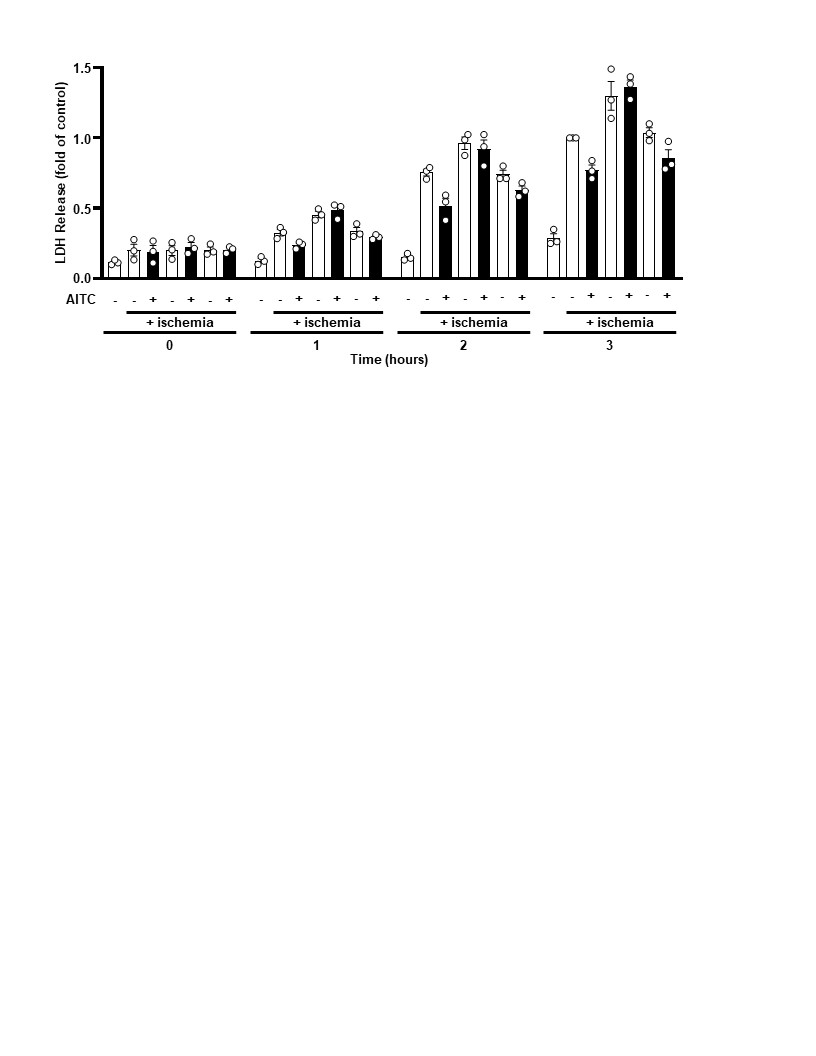

Supplement: Supplemental Material [file kchl-13-01-1623591-s001.zip › A1NO_SuppFig2.jpg]

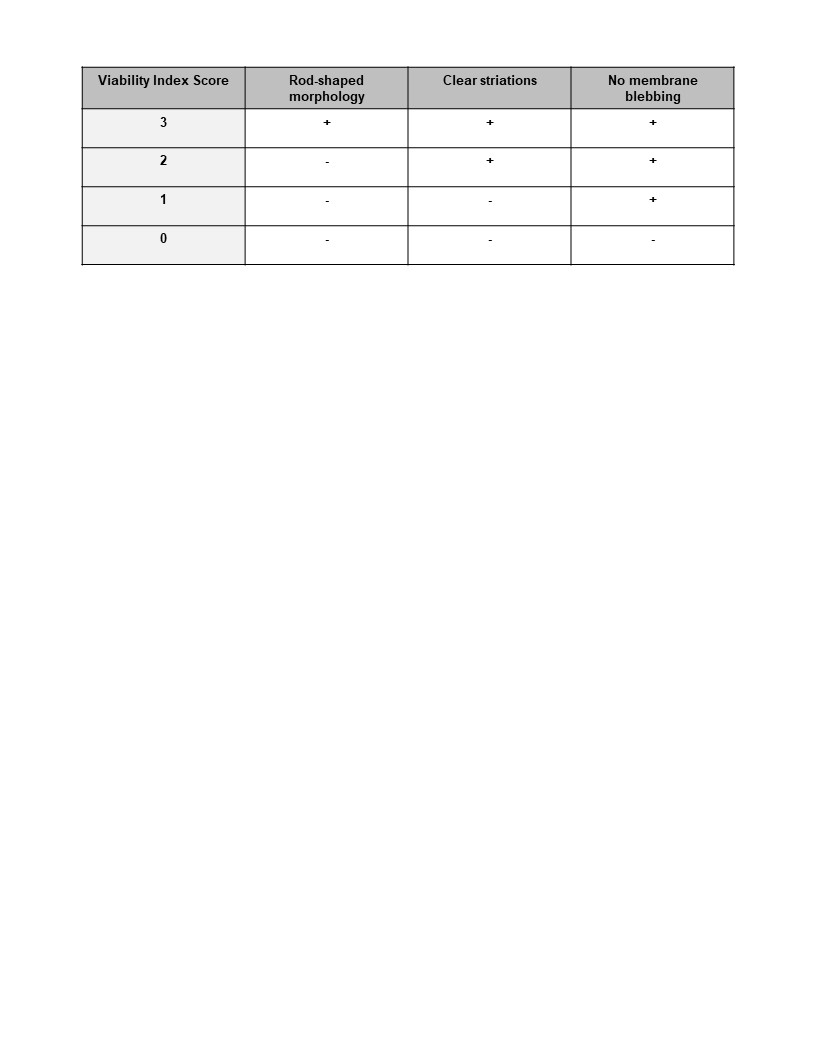

Supplement: Supplemental Material [file kchl-13-01-1623591-s001.zip › A1NO_SuppTable1.jpg]
